# An RFID-Based Self-Biased 40 nm Low Power LDO Regulator for IoT Applications

**DOI:** 10.3390/mi12040396

**Published:** 2021-04-03

**Authors:** Asghar Bahramali, Marisa Lopez-Vallejo

**Affiliations:** IPTC, ETSI Telecomunicación, Universidad Politécnica de Madrid, Avda. Complutense 30, 28040 Madrid, Spain; marisa@die.upm.es

**Keywords:** regulator, dropout voltage, quiescent current, RFID, IoT

## Abstract

There are emerging applications, like bridge structural health monitoring, continuous patient condition and outdoor aiding of the elderly and the disabled, where Internet of things (IoT) nodes are used with very limited accessibility and no connection to the main supply network. They may also be exposed to harsh environmental conditions. These are applications where power and available area constraints are of great concern. In this paper, we design a 1.1 V low dropout (LDO) linear regulator in 40 nm technology to be embedded in IoT nodes. To address these constraints, we used state-of-the-art, variability-aware resistor-less sub-threshold biased CMOS-only ultra low power consumption configurations having low active area. The proposed LDO is internally compensated with embedded 18 pF Miller and 10 pF load capacitances. It can supply 1 mA maximum load current with 0.8 uA quiescent current. The dropout voltage of the regulator is 200 mV with minimum input voltage of 1.3 V. The efficiency of the regulator is 84%, which is about 99% of the maximum achievable efficiency for a 200 mV dropout voltage. The whole circuit, consisting of the embedded voltage reference and the Miller and load capacitances, takes less than 0.007 $mm^{2}$ of the die size with 1 μW power consumption.

## 1. Introduction

Internet of things (IoT) applications suffer from power constraints because there is limited or no access to the main supply, especially when the nodes are placed at inaccessible locations or in harsh environments. Using a local battery can be considered as an easy and fast solution to this problem. However, today, for widely used IoT applications, where hundreds or even thousand of nodes may be available, the need to change the battery or recharging it imposes a high maintenance cost [1]. The problem gets exacerbated in case of outdoor applications as bridge health monitoring [2], where continuous access to the nodes is harmful and should be done through special precautions that again add up to the maintenance cost. Promising developments on different techniques have allowed to harvest energy from light, heat, vibration or electromagnetic radiation, to name a few [3]. RF waves are a particular case of electromagnetic radiation, which may be harvested by a Radio Frequency Identification (RFID) node [4].

RFID technology has reached a sufficient level of maturity to provide part of the physical layer of the IoT for multiple applications through low-cost and energy-autonomous sensors [5]. The supply voltage for the transponder’s circuits can be obtained by means of a power-efficient rectifier that gets the energy from the RF field [6]. However, this approach suffers from two main problems. First, only some micro-watts of power are available, which must be enough for the whole transponder chip. Second, severeuctuations may affect the rectified signal. A better option is to use a voltage regulator to provide a more stable supply voltage, and additionally reduce the ripple on it [7]. With this approach, the back-end circuits in the node are not affected much from the fluctuations of the supply voltage and the load current.

**Figure 1:**
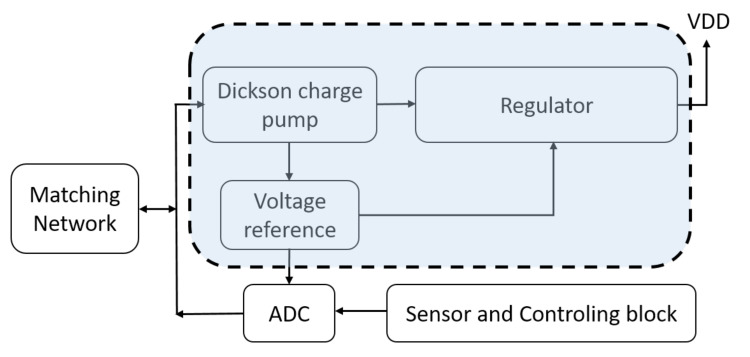
A typical power harvested platform.

In this paper, we propose a low dropout (LDO) linear regulator that produces 1.1 V DC voltage implemented with a 40 nm commercial technology. Special focus has been put on designing a regulator with very low power and low area, to allow room for other sub-circuits in the node. As is depicted in Figure 1, the regulator is part of the power unit of the IoT node. A Dickson charge pump which receives an 800 mV, 915 MHz harvested signal produces a 1.3 V DC voltage as supply voltage for the regulator. There is also a voltage reference, which is embedded in the LDO circuit that provides the comparison task for the regulation action. The whole configuration is self supplied and can be integrated with the targeted IoT node. The regulated voltage can supply a maximum current of 1 mA to the load with a quiescent current of 0.8 μA. The whole configuration consumes about 1 μW and takes about 0.007 $mm^{2}$ of the die size.

The paper is organized as follows: In Section 2, the concept of an LDO linear regulator with its related terms and parameters is reviewed. Section 3 depicts the challenges that the designers face in designing LDO regulators. In Section 4, a few of recently related reported work are reviewed. Section 5 introduces our proposed circuit configuration. In Section 6, the detailed results of the circuit simulation are provided, and Section 7 concludes our work.

## 2. LDO Linear Regulator Design Foundations

**Figure 2:**
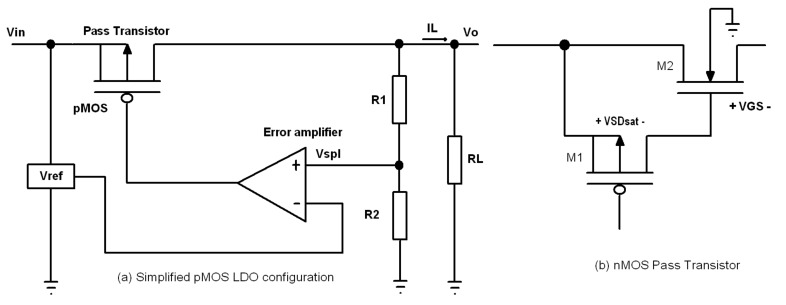
Simplified block diagram of a general linear regulator. (**a**) low dropout (LDO) linear regulator (having pMOS pass transistor). (**b**) a typical configuration of an nMOS pass transistor.

A simplified block diagram of a general linear regulator is shown in Figure 2a. As it uses a pMOS device as a pass transistor, it is called a low dropout (LDO) linear regulator.

Accuracy and efficiency are the main characteristics which are expected from voltage regulators for all intended applications [8]. Efficiency is assessed in terms of the quiescent current ($I_{Q}$) and the dropout voltage. The quiescent (or ground) current is the static current that the regulator needs to perform its regulating action. The dropout voltage is the lowest possible headroom voltage that is needed to drop across the drain-source of the pass transistor to regulate the output voltage and not to enter to its ohmic region of operation. Accuracy is mainly expressed in terms of load regulation, line regulation, and the ability of the regulator to resist temperature and process variations [9]. Power supply rejection and noise confinement are also among the desired qualities that reflect the accuracy of voltage regulators. Fast transit time is another feature needed in many applications. This is the minimum time that the regulator takes to stabilize its output voltage upon the load change.

An nMOS device can be also used as a pass transistor with a common drain configuration, presenting very low output resistance. In this manner, it acts as a buffer having a high current gain that can feed applications that demand great load changes. With this configuration, the pole related to the output node will be located very far from the pole produced by the gate of the pass transistor. This leads to a dominant pole configuration with little stability issues. The high mobility of electron carriers in nMOS devices allows lower device size for a specific current [10]. Despite these features, they suffer from a few problems among them the most important ones are as follows:

- The common drain configuration provides a voltage gain of lower than one for nMOS pass transistors. It means that their gate node voltage should be at least equal to the output voltage. This can be done either by configurations, like the one shown in Figure 2b, or by a charge pump that provides the necessary voltage for this node. The former solution increases the voltage drop across the drain-source of the pass transistor which leads to more power consumption. The latter approach produces ripple in the gate of the device that can be reflected in the output voltage by the nMOS pass transistor. This degrades the power supply ripple rejection ratio (PSRR) performance of the regulator. To avoid this problem, large capacitances or RC filters may be used at the output of a charge pump that slow down the controlling action of the error amplifier and increase the transient response of the regulator [11]. As charge pumps are area consuming, it does not make any sense to use nMOS devices as pass transistors together with a charge pump, unless at least their total size is less than or equal to that of the pMOS pass transistor [12]. Using a charge pump also degrades the power efficiency of the circuit as its efficiency is not high.
- In common drain configuration of nMOS, the source and the body of the nMOS pass transistor are not at the same potential and the body effect appears. To fix this problem, a twin well nMOS device should be used, that is not provided by all foundries.
- nMOS pass transistors that have a source follower configuration in linear regulators do not show acceptable noise performance. Any noise which appears at the output of the error amplifier (the gate of the pass transistor) is transferred to the output node through the gate-source of the pass transistor.
- When a load variation initiates a current step, large ripple appears at the output. To deliver high currents to the load, a large nMOS pass transistor that has large gate-source capacitance should be used. This ripple couples through $C_{gs}$ to the gate of the pass transistor and causes big overshoots/undershoots at the gate voltage. A large capacitor to the gate of the device is needed to attenuate them [13]. Increasing the capacitance of the gate of the pass transistor pushes the dominant pole to a lower frequency and decreases the unity gain bandwidth of the regulator. This results in an increase of the response time of the feedback loop and consequently the transit time and the transient response of the regulator. Low unity gain bandwidth also degrades PSRR of the regulator when the frequency increases.

A pMOS pass transistor, in comparison with its nMOS countrpart, needs lower dropout voltage, it has lower threshold voltage without the body effect problem, its 1/*f* noise performance is better, and it shows higher power efficiency performance. These are the features that make an LDO linear regulator a true fit for our target applications. But, because of its common source configuration in the regulator, it has higher output resistance, which creates stability issues and degrades the load regulation performance of the regulator.

Next, we review some of the main regulator characteristics that are used in their assessment.

### 2.1. Efficiency

A key parameter of a regulator is efficiency (η), which is the ratio of the input power ($P_{IN}$) that is delivered to the output load [14]. Considering the variables in Figure 2a, it is defined by:

(1) $$\begin{array}{cc} \eta =\frac{P_{O}}{P_{IN}}\; & =\frac{I_{L}V_{O}}{\left(I_{L}+I_{Q}\right)V_{IN}}=\frac{I_{L}}{\left(I_{L}+I_{Q}\right)}\times \frac{V_{O}}{\left(V_{O}+V_{H}\right)}=\frac{1}{1+\frac{I_{Q}}{I_{L}}}\times \frac{1}{1+\frac{V_{H}}{V_{O}}}. \end{array}$$

As Equation (1) shows, the efficiency of the regulator increases when the quiescent current ($I_{Q}$) and the headroom voltage ($V_{H}$) are kept low with respect to the load current and the output voltage, respectively.

### 2.2. Analytical Model of the LDO

In an LDO, the $V_{SD}$ of the pMOS pass transistor is kept as low as possible. The regulator can regulate the output voltage as long as its pass transistor does not enter into its linear region of operation where $V_{SD_{pass}}<V_{SG_{pass}}-\left|V_{th_{pass}}\right|$. So, as $V_{SD_{pass}}$ has a low voltage value, its $V_{SG}$ should also be kept very low to prevent it from entering into this region of operation. When $V_{SG_{pass}}\approx \left|V_{th_{pass}}\right|$ ($V_{SG_{pass}}\leq \left|V_{th_{pass}}\right|$), the pMOS pass transistor enters into its sub-threshold region of operation. In this region, if $V_{SD}\geq 100\;mV$, the I/V characteristic of the pMOS transistor follows Equation (2):

(2) $$\begin{array}{cc} \;I_{SD}=I_{s}\exp\left(\frac{V_{SG}-\left|V_{th}\right|}{nV_{T}}\right)\times \left(1+\lambda V_{SD}\right),\;I_{s} & =\mu C_{ox}\frac{W}{L}\left(n-1\right){V_{T}}^{2}, \end{array}$$

where $V_{th}$ is the threshold voltage, *n* represents the sub-threshold slope factor, $V_{T}$ corresponds to the thermal voltage, λ denotes the coefficient of the channel length modulation, μ stands for the free carriers mobility, and $C_{ox}$ is the gate oxide capacitance.

With the assumption that the load current is much greater than the current taken by the sampling network and considering Equation (2), we can write from Figure 2a:

(3) $$\begin{array}{cc} I_{L} & =I_{s}\exp\left(\frac{V_{SG_{pass}}-\left|V_{th_{pass}}\right|}{nV_{T}}\right)\times \left(1+\lambda V_{SD_{pass}}\right)\\ \frac{I_{L}}{I_{s}}\times \frac{1}{(1+\lambda V_{SD_{pass}}}) & =\exp\left(\frac{V_{SG_{pass}}-\left|V_{th_{pass}}\right|}{nV_{T}}\right). \end{array}$$

In LDOs, the voltage drop across the pass transistor ($V_{SD_{pass}}$) is kept low. λ is also very low. Thus, we can say that $\frac{1}{1+\lambda V_{SD_{pass}}}\approx 1-\lambda V_{SD_{pass}}$ So, Equation (3) changes to:

(4) $$\begin{array}{cc} \frac{I_{L}}{I_{s}}\times \left(1-\lambda V_{SD_{pass}}\right) & =\exp\left(\frac{V_{SG_{pass}}-\left|V_{th_{pass}}\right|}{nV_{T}}\right)\\ V_{SG_{pass}}-\left|V_{th_{pass}}\right| & =nV_{T}ln\left(\frac{I_{L}}{I_{s}}\times \left(1-\lambda V_{SD_{pass}}\right)\right). \end{array}$$

As the result of the feedback action of the error amplifier (Figure 2a), we know that $V_{SG_{pass}}=V_{in}-A_{Oe}\left(V_{spl}-V_{ref}\right)$, where $A_{Oe}$ is the open loop gain of the error amplifier. So, Equation (4) changes to:

(5) $$\begin{array}{cc} V_{in} & -A_{Oe}\left(V_{spl}-V_{ref}\right)-\left|V_{th_{pass}}\right|=nV_{T}\ln\left(\frac{I_{L}}{I_{s}}\right)+nV_{T}ln\left(1-\lambda V_{SD_{pass}}\right). \end{array}$$

As $\lambda V_{SD_{pass}}\ll 1$, then $ln\left(1-\lambda V_{SD_{pass}}\right)\approx -\lambda V_{SD_{pass}}$, and $V_{SD_{pass}}=V_{in}-V_{O}$. So, we have:

(6) $$\begin{array}{cc} \;V_{in}-A_{Oe}\left(K_{spl}V_{O}-V_{ref}\right)-\left|V_{th_{pass}}\right|= & nV_{T}\ln\left(\frac{I_{L}}{I_{s}}\right)-nV_{T}\lambda \left(V_{in}-V_{O}\right)\\ \left(A_{Oe}K_{spl}+nV_{T}\lambda \right)V_{O}= & A_{Oe}V_{ref}+\left(1+nV_{T}\lambda \right)V_{in}-\left|V_{th_{pass}}\right|-nV_{T}\ln\left(\frac{I_{L}}{I_{s}}\right) \end{array}$$

where $K_{spl}$ is the sampling coefficient as follows:

(7) $$\begin{array}{c} K_{spl}=\frac{R_{2}}{R_{1}+R_{2}}, \end{array}$$

and $g_{m_{pass}}$ in the sub-threshold region of operation is defined as [15]:

(8) $$\begin{array}{c} g_{m_{pass}}=\frac{I_{L}}{nV_{T}}. \end{array}$$

We also know that the dynamic resistance seen from the drain-source of the pass transistor ($R_{DS_{pass}}$) in the sub-threshold region of operation is achieved by [15]:

(9) $$\begin{array}{c} R_{DS_{pass}}\approx \frac{1}{\lambda I_{L}}. \end{array}$$

From the Equations (8) and (9), we will have $nV_{T}\lambda =\frac{1}{g_{m_{pass}}R_{DS_{pass}}}$ that, if applied to Equation (6), changes to:

(10) $$\begin{array}{cc} V_{O}= & \left(\frac{A_{Oe}g_{m_{pass}}R_{DS_{pass}}}{1+K_{spl}A_{Oe}g_{m_{pass}}R_{DS_{pass}}}\right)V_{ref}+\left(\frac{1+g_{m_{pass}}R_{DS_{pass}}}{1+K_{spl}A_{Oe}g_{m_{pass}}R_{DS_{pass}}}\right)V_{in}\\ \; & -\left(\frac{g_{m_{pass}}R_{DS_{pass}}}{1+K_{spl}A_{Oe}g_{m_{pass}}R_{DS_{pass}}}\right)\left|V_{th_{pass}}\right|-\left(\frac{nV_{T}g_{m_{pass}}R_{DS_{pass}}}{1+K_{spl}A_{Oe}g_{m_{pass}}R_{DS_{pass}}}\right)\ln\left(\frac{I_{L}}{I_{s}}\right). \end{array}$$

As $g_{m_{pass}}R_{DS_{pass}}\gg 1$, Equation (10) changes to:

(11) $$\begin{array}{cc} V_{O} & \approx \left(\frac{1}{K_{spl}}\right)V_{ref}+\left(\frac{1}{A_{Oe}K_{spl}}\right)(V_{in}-|V_{th_{pass}}\left|\right)-\left(\frac{nV_{T}}{A_{Oe}K_{spl}}\right)\ln\left(\frac{I_{L}}{I_{s}}\right). \end{array}$$

Regarding Equation (11), the explicit effect of the variations of $V_{ref}$, $V_{in}$, $V_{th_{pass}}$, and $I_{L}$ in the output voltage is clear. Temperature and process variations can also affect the output voltage implicitly through $V_{T}$, $V_{th_{pass}}$, $A_{Oe}$, and $I_{s}$. Among these items, the effect of the variation of the voltage reference is more severe because there is no control from the feedback loop to restrict it.

The derived analytical model is used throughout this paper to size the components and to look for the trade-offs that should be made between contrasting performance parameters.

### 2.3. Stability

The common source configuration of the pMOS pass transistor in LDO linear regulators provides a high output resistance with the consequence of a low frequency pole at the output node of the regulator. The output node of the error amplifier also encounters the large capacitance of the gate-source of the pass transistor that together with other capacitances seen from this node are considered as a parasitic capacitance ($C_{par}$). This capacitance, in conjunction with its parallel total parasitic resistances ($R_{par}$), makes another low frequency pole. The effect of the output resistance of the pass transistor is worse in low load current as it is inversely proportional to it (Equation (9)).

Including a Miller capacitance across the drain and the gate pins of the pass transistor is an approach that helps to stabilize the regulator specially in applications where it is not possible to include off-chip large capacitors. It provides a negative feedback for the regulator that helps to stabilize the regulator. In this manner, any deviation of the output voltage from its desired value is fed back to the gate of the pass transistor so that its conduction is reversely proportional to the output voltage fluctuations. Therefore, the output voltage will be settled at its nominal value. The feedback gain is frequency dependent, so that, for very high frequencies, the pass transistor acts as a diode connected device having low output resistance. Thus, at high frequencies it remains not much stability issues due to the far output pole.

The Miller capacitance stabilizes the regulator at the expense of limiting the bandwidth. Lower bandwidth, although helps to stabilize the regulator and prevents the amount of injected noise, degrades the PSRR performance of the regulator. So, there should be a trade-off between these issues, although the stability has the greatest priority.

#### 2.3.1. Load Regulation

It is important that the regulator could maintain the nominal regulated voltage even if the load demand changes. This is called load regulation and represents the output resistance of the regulator ($R_{O_{reg}}$), which is related to the output resistance of the pass transistor $R_{SD_{pass}}$ through the feedback loop gain ($T=A_{O_{e}}g_{m_{pass}}R_{SD_{pass}}K_{spl}$). It is defined as [16]:

(12) $$\begin{array}{c} L_{OR}=R_{O_{reg}}=\frac{\Delta V_{O}}{\Delta I_{L}}\approx \frac{R_{SD_{pass}}}{1+T}\approx \frac{R_{SD_{pass}}}{1+A_{Oe}g_{m_{pass}}R_{SD_{pass}}K_{spl}}. \end{array}$$

The same result can be derived from the proposed analytical model (Equation (11)). Equation (12) shows that a circuit configuration with lower output resistance has better load regulation. As it is clear from Equation (12), load regulation is improved by having high $A_{Oe}$, $g_{m_{pass}}$ and $K_{spl}$. $K_{spl}$ is determined by the value of the voltage reference and should be regarded a constant value here. $g_{m_{pass}}$ in sub-threshold region of operation is defined by Equation (8). It is clear from this equation that, for a predefined load current, the value of $g_{m_{pass}}$ is fixed, and it cannot be increased any longer. Thus, the only way to improve the load regulation is by increasing the open loop gain of the error amplifier ($A_{Oe}$), which results in more power consumption.

#### 2.3.2. Line Regulation

The ability of the regulator to withstand against the variations originated from the supply voltage is expressed through the line regulation ($L_{iR}$) and power supply ripple rejection ratio (PSRR). For line regulation, the DC gain of the error amplifier is involved, and, for PSRR, its AC gain is taken into account. For regulators that have a dominant pole behavior, it is clear from the above definition that PSRR is equal to the line regulation for the frequencies below the cutoff frequency. It degrades constantly from this frequency up to the unity gain bandwidth frequency. Beyond that, the error amplifier loses its control over the output voltage regulation and it is the output capacitance that if selected correctly should be able to damp the output variation [17]. For example, for a typical regulator that has 80 of PSRR at 10 Hz, its PSRR can fall to as little as 20 at a few tens of kilohertz [18].

Line regulation and PSRR are defined as follows [19,20]:

(13) $$\begin{array}{c} L_{iR}=\frac{\Delta V_{O}}{\Delta V_{in}},\;PSRR=20log\left(\frac{V_{Out_{ripple}}}{V_{in_{ripple}}}\right). \end{array}$$

Line regulation can be improved by a high error amplifier open loop gain ($A_{Oe}$), which has the same effect on the load regulation.

The line regulation performance of the regulator can be studied by assuming a variable $R_{SD_{pass}}$ in place of the pass transistor in series with the load ($R_{L}$) [21] such that $V_{in}=\left(R_{SD_{pass}}+R_{L}\right)I_{L}$. With this assumption, we can write:

(14) $$\begin{array}{cc} L_{iR} & =\frac{\Delta V_{O}}{\Delta V_{in}}=\frac{\Delta V_{O}}{\Delta I_{L}}\frac{\Delta I_{L}}{\Delta V_{in}}=\frac{1}{\left(R_{SD_{pass}}+R_{L}\right)A_{Oe}g_{m_{pass}}K_{spl}}. \end{array}$$

As is clear from Equation (14), the line regulation is degraded by increasing the load current [18]. This effect is reflected in the line regulation both by decreasing $R_{SD}$ (Equation (9)) and the load resistance (to increase the load current). This shows that the regulator has an opposite behavior concerning its load and line regulation at high and low loads. So, a trade-off should be made between theses two performance parameters of the regulator.

### 2.4. Noise Analysis

Flicker (or 1/f) and channel thermal noises are two of the intrinsic noises that are generally considered as main sources of noise for CMOS devices [22]. Whenever resistors are used in the circuit their thermal noise also affects the desired output specially when their resistances are high.

Flicker noise has a process dependent characteristic that is less effective in LDO regulators due to the physical structure of the pMOS pass transistor [15]. In our design, we tried to restrict the thermal noise by designing resistor free configurations. So, the most effective noise will be the channel thermal noise of the devices, which is inversely proportional to the device’s current. In our work, where low power consumption of the regulator is of great importance, we shifted to the sub-micron technology to restrict the channel thermal noise. In this manner, we focused first on lowering the supply voltage and not lowering the circuit bias current. This results in both lower power consumption and better noise performance of the regulator.

### 2.5. Load Transient Response

The performance of a regulator is highly affected by the maximum change made in the output voltage under a transient time, which is called load transient response [23] or briefly transient response.

To have a smooth response, it seems inevitable to use capacitors at the output node of a linear regulator. They not only damp the ripples and provide stability but also supply the necessary current to the load when a rapid load change occurs. This helps the regulator to prevent overshoots/undershoots from appearing on the output voltage even before the controlling loop can show a reaction. Today, it is possible to embed on-chip capacitances up to 100 pF with the regulator inside the die. Capacitances larger than this amount are connected off-chip to the output node. External capacitors are not ideal and are influenced by temperature and output voltage variations. They have also parasitic resistance and inductance that can affect the performance of the regulator greatly. In our proposed circuit, an embedded 10 pF capacitor is regarded as the output capacitance load of the regulator. Metal-Oxide-Metal (MOM) capacitance can be used as an embedded capacitance. They have linear characteristics, but they are greatly under the influence of the process variations; and they show low capacitance, high series inductance and resistance; and they are prone to low breakdown voltage.

## 3. LDO Design Challenges

Stability, accuracy, and robustness against supply upstream and load downstream transients, temperature variation resiliency, low quiescent current, fast settling time, and low noise performance are features which are expected from a regulator. Since these are correlated parameters, there should be a trade-off among the expected specifications.

From the mathematical model of the regulator (Equation (11)) and the expressions derived for load regulation (Equation (12)) and line regulation (Equation1 (14)), it is obvious that the error amplifier open loop gain, the transconductance, and the output resistance of the pass transistor play an important role in achieving the required accuracy by an LDO. All these parameters, as well as the intrinsic noise performance of the regulator, are influenced by the load or the quiescent current. All of them except the output resistance of the pass transistor are improved by increasing these currents. The minimum accepted line regulation specifies the maximum output current and the maximum allowable power consumption limits the quiescent current. In defining these limits, the maximum acceptable noise at the output voltage also should be taken into account.

In a single pole system, if the open loop DC gain of the feedback loop increases while the dominant pole is kept constant, the unity gain bandwidth also increases. This may result in an inclusion of far poles in the bandwidth and jeopardizing the stability of the system. On the other hand, if the open loop DC gain of the feedback loop is decreased, it is possible that the left half plane zero, which is located in the bandwidth of the system to help its stability, is forced to the margin and destabilizes the system. Thus, from the stability point of view, an acceptable error amplifier open loop gain is the one which is neither too low nor too high.

## 4. Related Works

In Reference [24], mixed analog and digital techniques in 65 nm are used to produce a regulated output voltage of 0.45–0.95 V from the input voltage of 0.5–1 V. It uses a very large pMOS pass transistor to drive a maximum load of 100 mA. But this results in a very large capacitance at the gate of the pass transistor that produces stability issues. In this manner, an on chip large Miller capacitance of 40 pF is added to the gate-drain of the pass transistor, what results in a high active area of 0.04 $mm^{2}$.

In Reference [25], a 1 V regulated voltage is provided from an input range of 1.2–2.5 V with maximum load current of 100 mA. To drive this amount of the load current, a large pMOS pass transistor is used which produces stability problems. An output push pull stage that has low output impedance, as well as the bulk modulation technique, is used to overcome this problem. But this technique adds to the complexity and the fabrication cost of the circuit.

In Reference [26], a 2 V regulated voltage is produced in a 0.180 μm platform from an input range of 2.2–4 V with 70 μA quiescent current and the maximum load current of 50 mA. It uses a push pull buffer at the gate of the pMOS pass transistor to push the low frequency pole far away from the unity gain of the circuit. But the circuit power consumption due to the reported high quiescent current is high and not suitable for low power applications.

In Reference [27], 2.8 V regulated voltage is provided from a 3.3–3.5 V input voltage range. The circuit cascades an nMOS transistor with the pMOS pass transistor to improve the PSRR of the circuit. It can drive a 50 mA load at maximum with 50 μA quiescent current. But fairly high amount of minimum dropout voltage (0.5 V) with the high amount of the quiescent current result in high power consumption which makes the circuit not suitable for low power consumption.

In Reference [11], a back gate bias technique is used, which is available in 22FDX technology, to reduce the dropout voltage of the nMOS pass transistor. The circuit is devised with a charge pump and a ring oscillator as its clock to generate higher back-bias and reduce the threshold voltage of the nMOS pass transistor. Using a charge pump imposes more ripples to the circuit that need to be filtered by a filter which increases the die size. The proposed circuit introduces an output voltage of 0.91 V from an input voltage of 1.35 V with a load current capacity of 15 mA, which is achieved by a very large pass transistor. So, the dropout voltage of the regulator is 440 mV, which is not low. The circuit quiescent current is about 200 μA, which is not only very high but also provides a low ratio of $I_{L}/I_{Q}$. The high dropout voltage, as well as a high quiescent current, makes the circuit a power hungry configuration.

In Reference [28], an AB amplifier is implemented to regulate a 1 V voltage from an input voltage of 1.2 V. It can deliver ±80 μA to the load while consuming 1.8 μA quiescent current. The circuit is complicated and the chip active area is high. The stability of the circuit is achieved with a high total 100 pF capacitance that occupies a large amount of 0.24 $mm^{2}$ of the die area.

## 5. Proposed LDO Configuration

**Figure 3:**
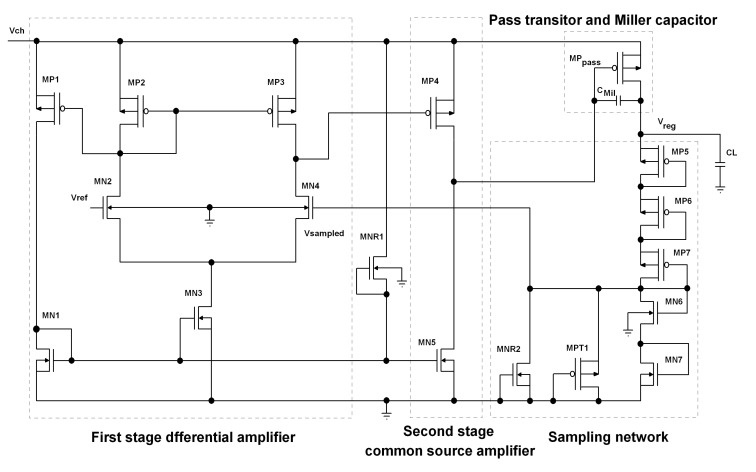
Simplified schematic of the presented LDO regulator.

In this paper, a capacitor-free LDO, based on the configuration shown in Figure 3, is designed. It consists of a two-stage amplifier, the pass transistor, the sampling network and the voltage reference circuit (which is not shown in this figure). Reverse diode connected devices, like MNR1 and MNR2, are also included in the circuit to compensate for the non-linear temperature behavior of the regulator. The whole regulator, including the voltage reference and the load capacitor is integrated in the same die to provide a fully integrated regulator. With on-chip capacitors, load transient voltage spikes that can stem from the parasitic inductance of the bond wires are removed, and the crosstalk phenomena is reduced [29].

In our design, we have not used resistors for the sampling network because resistances take much of the die size and become a great source of noise. As the difference between the sampled voltage and the voltage reference is amplified by the error amplifier and the pass transistor, it is critical to confine the noise from these blocks. The proposed configuration is designed such that it does not deteriorate the temperature behavior of the sampled voltage because it is possible to produce a positive coefficient current for the sampling network that can help to cancel out the negative coefficient behavior of the $V_{GS}$ of MN6 and MN7 devices (Figure 3). This is possible by the adjustment of the aspect ratios of the devices in the sampling network [30].

In our design, we have used a two-stage error amplifier (EA). In literature, EAs with two stages are more often used. In this manner, an acceptable open loop gain is provided, which is needed to fulfill various expected metrics of the regulator, such as stability [31]. The first stage is an nMOS input double-to-single-ended differential amplifier, which is cascaded with a pMOS common source amplifier (Figure 3). This configuration has proved to have better line regulation and PSRR performance among the other possible configurations [31].

The pMOS common source configuration (MP4) also provides a high positive saturation (≈$V_{DD}-V_{SD_{sat}}$) for the error amplifier. This is necessary specially in case that an instantaneous decrease in load occurs. In this condition, if the source-gate of the pass transistor is not decreased, the capacitors which are present at the output node will charge to a voltage higher than the regulated one, and a large overshoot may happen at the output voltage [32].

We have made use of an 18 pF Miller capacitance between the drain and the gate pins of the pass transistor to stabilize the feedback loop. We have also used an embedded 10 pF capacitance as the load capacitance in our design.

### Temperature Model of the Proposed Regulator

The temperature coefficient (TC) of the output voltage of a circuit is defined as:

(15) $$\begin{array}{c} TC=\frac{1}{V_{O_{Nominal}}}\frac{\Delta V_{O}}{\Delta T}, \end{array}$$

where $V_{O_{Nominal}}$ is the output voltage at 27 ${}^{{}^{\circ}}C$, and T is the absolute temperature.

It is not easy to provide high temperature resiliency for this design because we cannot take advantage of the resistors’ positive temperature coefficient that can be used to cancel the negative temperature coefficient of $V_{GS}$ of the CMOS devices. Our design, where the whole platform is supplied by a Dickson charge pump, benefits from the positive temperature coefficient of the charge pump as a substitute for the bulky resistors from this point of view. This is a new concept that first introduced in [30] and followed in [33,34].

By looking at the mathematical model that we have derived by Equation (11), there are various parameters that shape the temperature performance of the proposed regulator. To simplify our analysis, we assume that $\frac{\partial k_{spl}}{\partial T}=0$, which is done through the combination of the MPT1 and MNR2 devices. $A_{Oe}$, which is the open loop gain of the error amplifier, is comprised of a differential amplifier and a common source stage (Figure 3). So, $1/A_{Oe}$ at its minimum value can be derived as:

(16) $$\begin{array}{cc} \frac{1}{A_{Oe}} & =\frac{1}{2g_{m_{MN2}}R_{DS_{MP3}}g_{m_{MP4}}R_{DS_{MN5}}}=\frac{1}{2}{\left(n\lambda V_{T}\right)}^{2}. \end{array}$$

Thus, its temperature coefficient is equal to $\frac{\partial (\frac{1}{A_{Oe}})}{\partial T}=\frac{{\left(n\lambda V_{T}\right)}^{2}}{T}$, which has a small value, and we will neglect it in the following relations.

Considering Equation (11), we can derive the temperature coefficient of the proposed regulated voltage as follows:

(17) $$\begin{array}{cc} \frac{\partial V_{O}}{\partial T} & \approx \left(\frac{1}{K_{spl}}\right)\frac{\partial V_{ref}}{\partial T}+\left(\frac{1}{A_{Oe}K_{spl}}\right)\left(\frac{\partial V_{in}}{\partial T}-\frac{\partial V_{th_{pass}}}{\partial T}\right)-\left(\frac{nV_{T}}{A_{Oe}K_{spl}}\right)\left(\frac{1}{T}\ln\left(\frac{I_{L}}{I_{s}}\right)+\frac{1}{I_{L}}\frac{\partial I_{L}}{\partial T}-\frac{1}{I_{s}}\frac{\partial I_{s}}{\partial T}\right). \end{array}$$

By substituting $A_{Oe}$ with its equivalent from Equations (16) and (17), it changes to:

(18) $$\begin{array}{cc} \frac{\partial V_{O}}{\partial T} & \approx \left(\frac{1}{K_{spl}}\right)\frac{\partial V_{ref}}{\partial T}+\left(\frac{{\left(n\lambda V_{T}\right)}^{2}}{2K_{spl}}\right)\left(\frac{\partial V_{in}}{\partial T}-\frac{\partial V_{th_{pass}}}{\partial T}\right)-\left(\frac{\lambda^{2}{\left(nV_{T}\right)}^{3}}{2K_{spl}}\right)\left(\frac{1}{T}\ln\left(\frac{I_{L}}{I_{s}}\right)+\frac{1}{I_{L}}\frac{\partial I_{L}}{\partial T}-\frac{1}{I_{s}}\frac{\partial I_{s}}{\partial T}\right). \end{array}$$

According to Equation (2), if we consider $\mu =\mu_{0}T^{\left(-m\right)}$, then $\frac{1}{I_{s}}\frac{\partial I_{s}}{\partial T}=\frac{2-m}{T}$. By substituting it in Equation (18) and assuming that the load current is not changed with the temperature, we will have:

(19) $$\begin{array}{cc} \frac{\partial V_{O}}{\partial T} & \approx \left(\frac{1}{K_{spl}}\right)\frac{\partial V_{ref}}{\partial T}+\left(\frac{{\left(n\lambda V_{T}\right)}^{2}}{2K_{spl}}\right)\left(\frac{\partial V_{in}}{\partial T}-\frac{\partial V_{th_{pass}}}{\partial T}\right)-\left(\frac{\lambda^{2}{\left(nV_{T}\right)}^{3}}{2TK_{spl}}\right)\left(\ln\left(\frac{I_{L}}{I_{s}}\right)+m-2\right). \end{array}$$

As is clear from Equation (19), the temperature behavior of the regulator is greatly under the influence of the temperature behavior of the voltage reference. We used the proportional to absolute temperature (PTAT) behavior of the output voltage of the Dickson charge pump to help to compensate the complementary to absolute temperature (CTAT) characteristics of the gate-source and the threshold voltages of the CMOS devices. The existence of $T^{2}$ and logarithm in the analytical model of the temperature behavior of the regulator is an indication of its nonlinear behavior. This makes it difficult to cancel the temperature variation effect on the regulated voltage.

## 6. Simulation Results

The circuit is simulated using Cadence spectre. In these reported results, the parasitic effects of the layout are also included through the Calibre extraction tools.

The circuit is assumed to be supplied by a Dickson charge pump that produces 1.3 V with 40 mV pp ripple at its output. The regulator is designed to provide 1.1 V regulated voltage, which is the nominal voltage for the back end circuitry in the platform that the devices in our target 40 nm technology are designed for.

In our simulation, the focus has been on 110 μA as the nominal load and 1.1 μA as the minimal one. The reason for choosing this amount of the nominal load is that, at this current, the devices are kept in the sub-threshold region of operation to save power [35].

### 6.1. Quiescent Current, Dropout Voltage, and Efficiency

The presented regulator drags an average current of 110.8 μA from the supply voltage at the nominal load. So, the whole circuit configuration needs 800 nA quiescent current to regulate the output voltage. This means that the regulator quiescent current is 0.7 of its (nominal) load current, which is low enough for low power applications. From this amount, 151.1 nA are devoted to the voltage reference circuit. The quiescent current for the minimum load is 854 nA. This is due to the fact that for low load the output resistance of the pass transistor is high, thus the error amplifier needs more gain to keep the output voltage regulated.

The circuit is designed to regulate 1.1VDC from 1.3 voltage input source. This results in a 200 mV voltage drop across the source drain of the pass transistor, which is among the lowest possible dropout voltage reported in the literature.

The efficiency of the whole configuration of the regulator (η) is, according to Equation (1), equal to 84, which is very close to the maximum efficiency (85) that can be achieved from an LDO that regulates 1.1 V output voltage with 200 mV dropout voltage.

### 6.2. Load Regulation Performance

**Figure 4:**
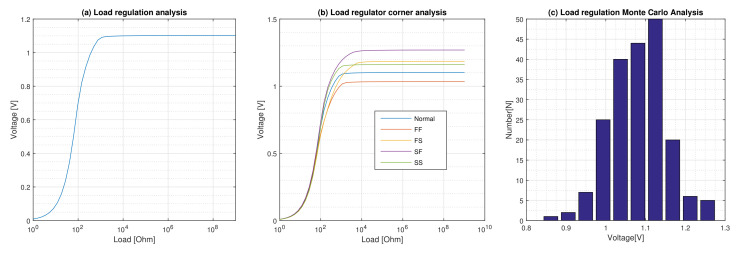
Load regulation performance of the regulator. (**a**) Single load regulation analysis. (**b**) Load regulation corner analysis. (**c**) Load regulation Monte Carlo analysis with 200-run.

We achieved a wide range load regulation so that the proposed regulator can regulate the output voltage from 1.085 V at 1 mA up to 1.102 V at 1 nA. This means that there is only 1.5 fluctuation from the nominal 1.1 V in this wide range of load variation. This is shown in Figure 4a. This figure, for our nominal and minimal loads range, is less than 0.2 that results in $L_{OR}$ = 20 Ω according to the definition from Equation (12).

The results of the corner and Monte Carlo simulation analysis for load regulation are shown in Figure 4b,c, respectively. The effect of process variations is analyzed by using worst typical and best case models of components. Usually, combinations of fast (F) and slow (S) nMOS and pMOS devices are considered creating four extreme corners of operation for the circuit: FF, FS, SF, and SS. The result of the process variations on the load regulation of the regulator for the nominal load is fluctuations of −7, +15.5. This result is achieved without applying trimming.

**Figure 5:**
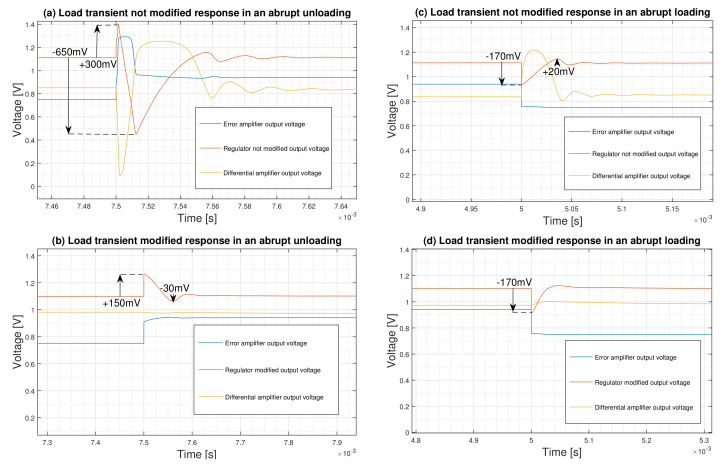
Load transient response analysis in abrupt load changes from the nominal load to the minimum load, and vice versa.

The reactions of the regulator in abrupt load change are shown in Figure 5. An abrupt load change with 10 ns rise and fall time, specially from nominal load to the minimum one, results in considerable fluctuations of +300 mV and −650 mV from the nominal 1.1 V regulated voltage and ringing (Figure 5a). This results in a transient response of $\Delta V_{tr_{max}}$ = 950 mV, which is very high and unacceptable. By modifying the aspect ratio of the MP4 device, the regulator could confine this figure to $\Delta V_{tr_{max}}$ = 180 mV (+150 mV and −30 mV) as is shown in Figure 5b. For a loading condition where fluctuations span between −170 mV and +20 mV ($V_{tr_{max}}=190\;mV$), the role of the proposed modified circuit is to provide a soft response removing overshoots and ringing (Figure 5c,d).

**Figure 6:**
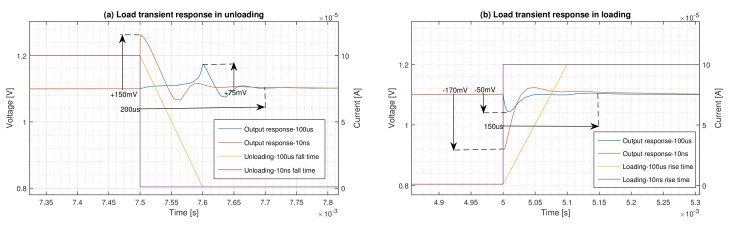
Load transient performance comparison between abrupt load change ($t_{r}=t_{f}=10ns$) and moderate load change ($t_{r}=t_{f}=100$μs).

The performance of the regulator in abrupt load changes with 10 ns rise and fall times is compared with that of the moderate load changes with 100 μs rise and fall times in Figure 6. As is shown in Figure 6a, the overshoot is confined for the unloading case from +150 mV to +75 mV. It is clear from Figure 6b that the undershoot for loading condition is reduced from −170 mV to −50 mV, and the ringing is completely disappeared. This results in $\Delta V_{tr_{max}}=105mV$ and $\Delta V_{tr_{max}}=50\;mV$ for moderate unloading and loading condition, respectively.

**Figure 7:**
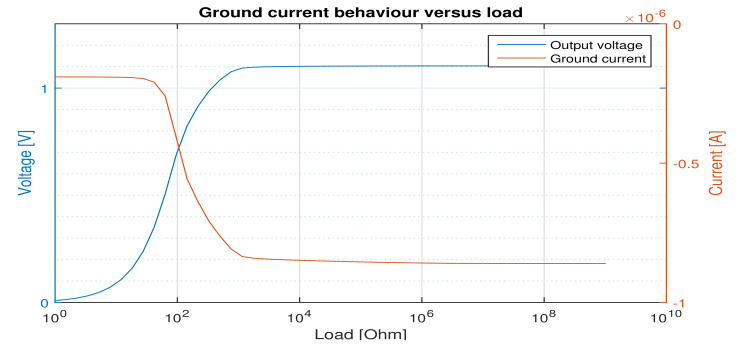
Ground current behavior versus load current.

The quiescent (ground) current of the whole regulator including the embedded voltage reference decreases by increasing the load current (Figure 7). As far as the regulator can perform its regulation task, the ground current decrease (from 854 nA to 800 nA) is not sensible from the minimum to the nominal load. But, when there is no regulation, it decreases rapidly to 100 nA. This is due to the error amplifier configuration that is used in our work. When higher load current is needed the second stage of the error amplifier conducts less current to increase the $V_{SG_{pass}}$. In this manner, less current is drawn from the supply source.

### 6.3. Transient, Settling and Start-up Time

The transient time of the regulator when a load transient happens can be evaluated from Figure 6. According to this figure, the transient times are 200 μs and 150 μs for abrupt unloading and loading conditions, respectively. The settling time for the regulator to reach to 99 of its regulated value is half of its transient time.

**Figure 8:**
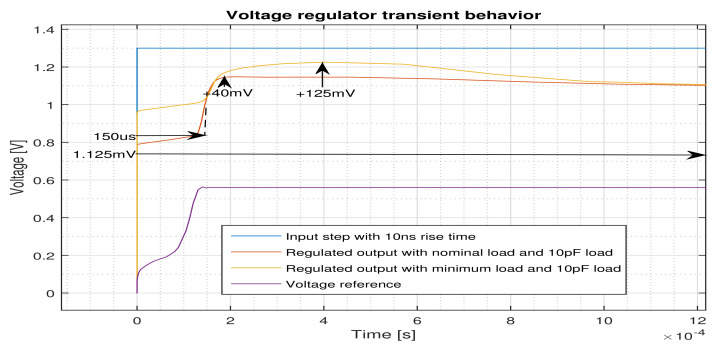
Input step response of the regulator for the nominal and minimum loads.

The step response of the regulator with 10 ns rise time both for the nominal and the minimum load cases is shown in Figure 8. With an input rise time of 10 ns, it takes about 150 μs for the regulator to reach to 90 of its regulated value for the nominal load. This is called the start-up time, and, as a matter of fact, this is the time that the reference circuit needs to provide the voltage reference for the regulator. In outdoor applications, where, in order to save energy, the device may be frequently switched on and off, a low start-up time becomes an important feature of the regulator [17]. The time that it takes for the regulator to provide the regulated voltage for both load conditions is around 1.125 ms. The output voltage is reached to its nominal value very softly after having a peak voltage of 40 mV and 125 mV for the nominal and minimum load conditions, respectively, without suffering from ringing.

### 6.4. Line Regulation Performance

**Figure 9:**
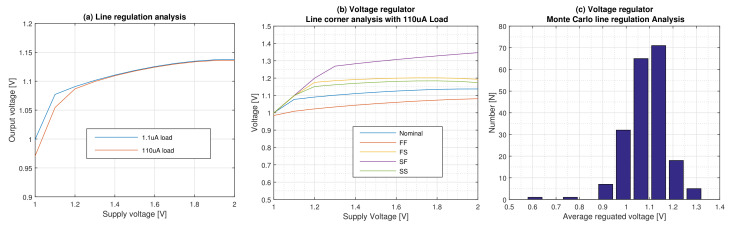
Line regulation performance of the regulator. (**a**) Line regulation for both nominal and minimum loads. (**b**) Line regulation corner analysis for the nominal load. (**c**) 200-run line regulation Monte Carlo analysis for the nominal load.

The line regulation of the proposed regulator is shown in Figure 9a. It is clear from this figure that, for both nominal and minimum loads conditions, the regulator can regulate 1.1 V at 1.3 V input voltage, and it can provide 5.7 regulation up to 2 V input voltage. This means that the dropout voltage at 1.3 V input voltage is 200 mV.

The line regulation performance of the regulator on the four corners is shown in Figure 9b and the results are supported with the Monte Carlo analysis for 200 runs (Figure 9c). The variations of the regulated voltage from its nominal value due to process variations are −5.5, +15.5 and −5.8, +14 at both nominal and minimum loads respectively without trimming.

**Figure 10:**
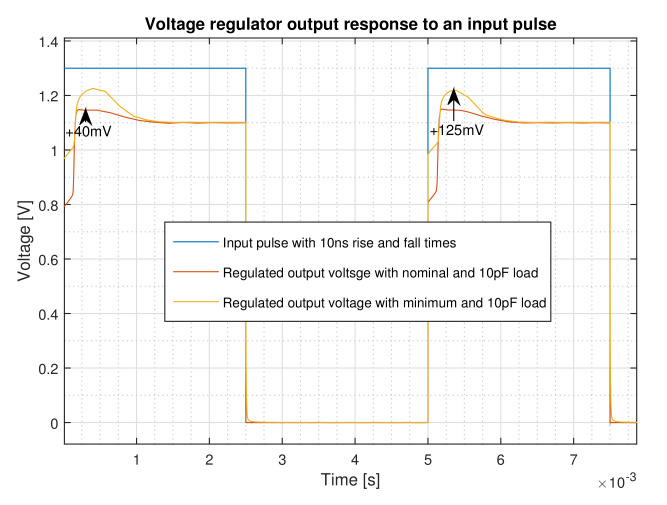
Output voltage response to an input pulse with 10 ns rise and fall times for both nominal and minimum loads.

The performance of the circuit against the abrupt source voltage changes for both nominal and minimal loads are shown in Figure 10. The circuit provides a stable regulated voltage when an abrupt supply voltage change from 0 to 1.3V is imposed. In this condition, the circuit experiences only 40 mV overshoot at nominal loading without showing any oscillation or ringing. This figure for the minimum loading is 125 mV.

The regulator peaks include only 1.5 $mV_{pp}$ ripple from the 40 $mV_{pp}$ ripple that appear at the output of Dickson charge pump. This results in PSRR of −28.5 @915 MHz with the nominal load. For the minimum load, PSRR becomes −34.5 @915 MHz.

**Figure 11:**
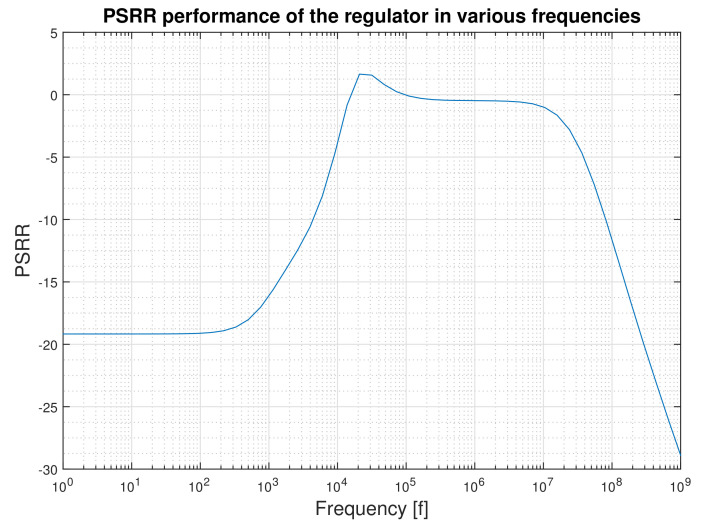
Power supply ripple rejection ratio (PSRR) performance of the regulator in various frequencies for the nominal load.

If we look at the PSRR curve of the regulator with respect to the frequency (Figure 11) three distinct regions can be recognized. The first region is at low frequencies up to the unity gain band width (12.227 kHz) that the regulator has the control on regulating the input voltage. After this frequency up to nearly 1 MHz not the error amplifier nor the output capacitance have control on the supply regulation. After this frequency the output capacitor has the key role in suppressing the input ripple. This is achieved due to an embedded 10 pF load capacitance. It is clear that, with a larger output capacitance, better PSRR could be achieved, but this increases the transient time of the regulator and is in clear contrast to the low die size strategy that is sought by our target applications.

### 6.5. Noise Performance

**Figure 12:**
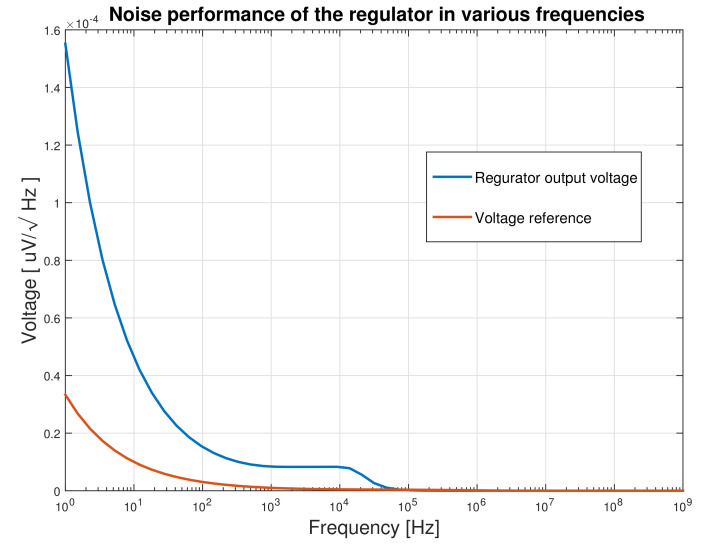
Noise density of the regulator output and the embedded voltage reference.

The noise density of both the regulator and the embedded voltage reference outputs, for the range of frequency of up to 1 GHz and for the nominal load is depicted in Figure 12. In the proposed regulator, the dominant noise is made of 1/f noise [36] with a low corner frequency of $f_{c}=42$Hz. Then, the white noise dominates up to 100 kHz that its value is 515 $nV/\sqrt{\mathrm{Hz}}$. The integral noise of the regulator for the frequency range of 10 Hz to 1 GHz are 1.268 mV and 1.528 mV for the nominal and the minimum loads, respectively. No noise peaking is seen near the unity gain bandwidth of the regulator, which is due to the suitable phase margin of the circuit.

### 6.6. Temperature Performance

**Figure 13:**
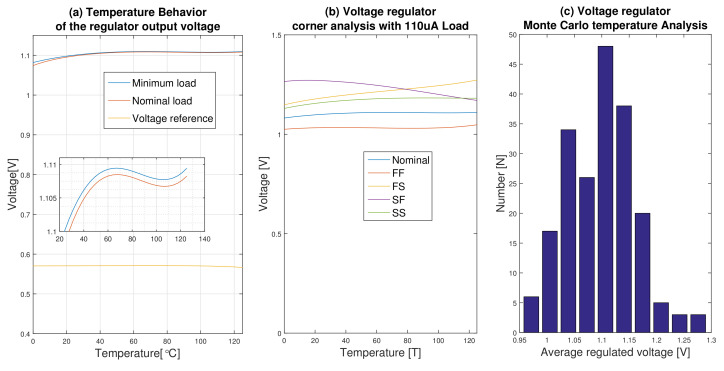
Temperature variation analysis. (**a**) Temperature analysis of the regulator for both nominal and minimum loads and the embedded voltage reference. (**b**) Temperature behavior corner analysis of the regulator. (**c**) 200-run Monte Carlo analysis of the temperature behavior of the regulator.

The behavior of the regulated voltage under temperature variation for both the nominal and the minimum loads is shown in Figure 13a. In this figure, the temperature behavior of the embedded voltage reference is also shown. The temperature coefficient (TC) of the voltage reference is 40 PPM/${}^{{}^{\circ}}C$ in a temperature range of −55 to 125 ${}^{{}^{\circ}}C$. As can be seen in this figure, the load current has not much effect on the regulator output voltage when the temperature rises. But at low temperatures more voltage drops occur at high currents. The TC of the proposed regulator is equal to 91PPM/${}^{{}^{\circ}}C$ in a temperature range of 25 to 125 ${}^{{}^{\circ}}C$. The corner analysis of the circuit for the nominal load shows −6.4 and +14.5 and for the minimum load shows −5.5 and +15.4 deviations from the nominal value due to process variations (Figure 13b). The result of the Monte Carlo analysis for the average value of the regulated voltage is also shown in Figure 13c for the nominal load.

### 6.7. Stability Performance

**Figure 14:**
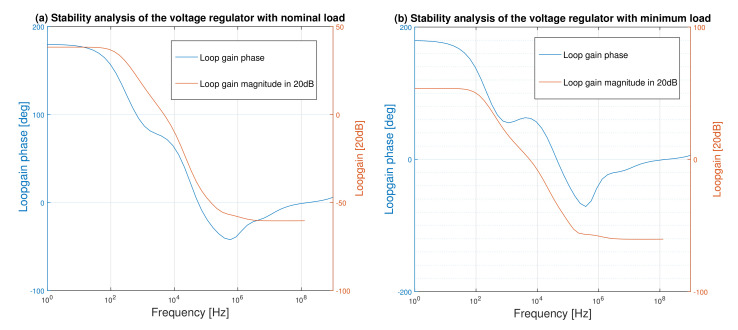
Bode plot stability analysis of the voltage regulator. (**a**) Nominal load. (**b**) Minimum load.

The stability performance of the regulator in the form of the loop gain and the loop phase is shown in Figure 14. As is shown in this figure, the regulator is stabilized with a phase margin of 58${}^{{}^{\circ}}$ at 12.22 kHz and a gain margin of 20.51 at 55.44 kHz at the nominal load. At the minimum load that the output resistance of the pass transistor increases the regulator has an acceptable phase margin of 46${}^{{}^{\circ}}$ at 14.63 kHz with a gain margin of 14.29 at 42.77 kHz.

What is more important than the phase margin is its load and the line step and pulse transient behavior of the circuit, that can ensure the stability of the circuit and are shown in Figure 5, Figure 6, Figure 8, and Figure 10, respectively. These figures show that the regulator behaves well under these load and line conditions without showing any oscillation and ringing.

### 6.8. Layout Size

**Figure 15:**
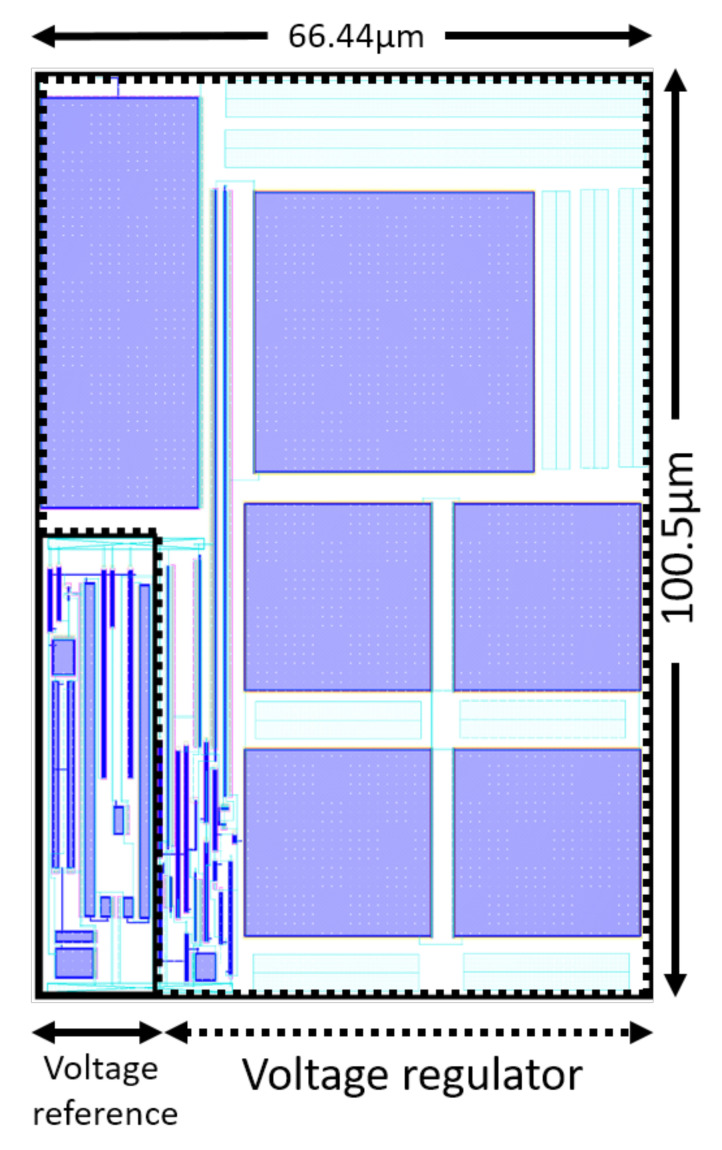
Layout of the regulator with the embedded voltage reference and the load and the Miller capacitances is 0.0067 $mm^{2}$.

Having a low area chip is one of our objectives to address the low area constraint of the target applications. The area of the layout of the whole regulator including the voltage reference and the load and the Miller capacitors (Figure 15) is:

$$\begin{array}{c} 100.5\;\mu m\times 66.44\;\mu m=0.0067\;mm^{2}. \end{array}$$

## 7. Conclusions and Discussion

In this work, we have presented an all embedded CMOS-only capacitor-less 1.1 V LDO regulator in 40 nm technology with the minimum feasible voltage drop of 200 mV across the pass transistor. The circuit is supplied by a Dickson charge pump that provides 1.3 V from an 800 mV, 915 MHz harvested voltage. The quiescent current of the whole configuration is 800 nA witch is 0.08 of the maximum 1 mA that can be delivered to the load while the output voltage is regulated. The efficiency of the regulator is 84. The proposed circuit is based on a standard two-stage amplifier which has the best line regulation among the possible available configurations. The circuit is designed with simple state of the art techniques using minimum device count to provide better performance regarding temperature resiliency and load transition response. Using the PTAT behavior of the Dickson charge pump to provide temperature stability is one of the features of our work. In this manner, a regulated voltage is provided with a TC of 91PPM/${}^{{}^{\circ}}C$ in a temperature range of 25 to 125 ${}^{{}^{\circ}}C$. The voltage fluctuation in an abrupt load transient is 180 mV–190 mV and is 105 mV and 50 mV with moderate unloading and loading transients, respectively. The circuit is stabilized using an 18 pF Miller capacitance across the pass transistor. This way, the regulator provides 1.1 V regulated voltage with 1.5 fluctuation from the minimum load to the maximum load (0.2 for the nominal load). The regulator provides 5.7 line regulation for an input range of 1.3 to 2 V. The circuit provides a PSRR of −28.5 @915 MHz at nominal load and −34.55 @915 MHz at minimum load. The circuit corner noise is 42 Hz with 515 $nV/\sqrt{\mathrm{Hz}}$ in 100 kHz. The integral noise of the regulator for the frequency range of 10 Hz to 1 GHz are 1.268 mV and 1.528 mV for the nominal and the minimum loads, respectively. All these figures are achieved with an embedded 10 pF load capacitance.

The key features of our proposed LDO compared with those extracted from the recently reported works are listed in Table 1. Considering the data presented in this table, our work has got FOM1 of 3.6 according to the definition of FOM1 as:

(20) $$\begin{array}{c} FOM1=\frac{C_{Total}V_{Drop}I_{Q}^{2}}{I_{L_{Max}}}. \end{array}$$

In this manner, our work was placed in the middle between those mentioned in this table. Providing a high $I_{L_{Max}}$, which is the key item for the reported works, to have a low FOM1 is not the objective of our work as the target applications should not consume much current. It is quite clear that a high load current can be provided by a large pass transistor which adds to the active area of the configuration.

In our configuration, if the load current increases, the quiescent current does not increase. So, if the low active area is not a constraint, it is possible to increase the maximum load current in our circuit and achieve a better FOM1. In order to have a better comparison between our work and the reported ones, we introduced FOM2 as follows:

(21) $$\begin{array}{c} FOM2=C_{Total}V_{Drop}I_{Q}. \end{array}$$

This is a FOM which consists of three important components which are expected to be as low as possible in any capacitor-less LDO regulator. Regarding FOM2, our work has got the second position among the reported works which are mentioned in Table 1. Realizing our circuit in 40 nm technology makes it possible to be integrated in a mixed mode with the digital circuits that are today implemented in deep nanometer technologies.

In the proposed design, an embedded voltage reference is used; although it makes the circuit independent from off-chip devices, it degrades its performance when compared with those mentioned in Table 1. In our work, achieving acceptable performance regarding the temperature resiliency and the intrinsic noise are also sought, which is not reported to be followed by the works in this table. This is not possible without compromising other metrics. So, there has been a trade-off between these items and other parameters, like load and line regulation.

## Figures and Tables

**Table 1:** Comparison between various aspects of the proposed circuit with recent published related works.

	This Work	Ref. [24]	Ref. [25]	Ref. [26]	Ref. [37]	Ref. [27]	Ref. [11]	Ref. [28]
Year		2019	2017	2019	2019	2018	2017	2018
Technology	40 nm	65 nm	0.18 μm	0.18 μm	0.18 μm	0.350	22FDX	0.18 μm
Pass Transistor	pMOS	pMOS	Push-pull	pMOS	pMOS	pMOS	nMOS	Push-pull
Active Area ($mm^{2}$)	0.0067	0.04	0.022	—	0.094	—	0.02	0.24
Quiescent Current (μA)	0.8	4.9	3.4	70	1.9	37.7	200	1.8
Maximum $I_{L}$ (mA)	1	105	∓100	50	100	50	15	∓80
Drop-out Voltage (mV)	200	50	200	200	200	500	440	200
Input voltage (V)	1.3–2	0.5–1	1.2–2.5	2.2–4	1.2	3.3	1.35–2	1.2
Regulated voltage (V)	1.1	0.45–0.95	1	2.2	1	2.8	0.77–0.91	1
Efficiency (%)	84.6	95	83.3	91.5	83.3	85	67	83.3
Phase margin	58${}^{{}^{\circ}}$	—	35-85	81${}^{{}^{\circ}}$	61.1${}^{{}^{\circ}}$	57 ${}^{{}^{\circ}}$	45${}^{{}^{\circ}}$	104${}^{{}^{\circ}}$
$C_{L}$ embedded (pF)	10	0	10	0–100 off-chip	—	10	30	100
$C_{Total}$ embedded (pF)	28	42	12.5	4	18	35	30	100
Transient voltage (mV)	190	88	220	290	54	—	63	227
PSRR (dB)	−28.5@915MHz	—	−49@1 Hz–20@10 kHz	—	—	−445@100 KHz	−35@10 MHz	−30@10 Hz@80 mA
FOM1 ($pF\times V\times \mu A^{2}/mA$)	3.6	0.48	0.29	78	0.13	497	35,200	0.81
FOM2 (pF×V×μA)	4.5	10.3	8.5	56	2.5	660	2640	36
